# Advanced human organoid-on-chip with physiological cellular complexity reveals bidirectional secretion patterns

**DOI:** 10.1016/j.isci.2025.114418

**Published:** 2025-12-15

**Authors:** Inga Viktoria Hensel, Szabolcs Éliás, Michelle Steinhauer, Claudia Günther, Martín Resnik-Docampo

**Affiliations:** 1 BioMed X Institute, Heidelberg 69120, Germany; 2 Department of Medicine 1, Friedrich-Alexander-University, 91054 Erlangen, Germany

**Keywords:** immunology, biological sciences, biomedical engineering

## Abstract

Intestinal epithelial cells are essential for maintaining gut homeostasis, serving as a physical barrier separating luminal content from the immune compartment. They are crucial for balancing tolerance to commensal and protection from pathogenic bacteria and dietary antigens by the asymmetric secretion of immune regulators. To understand how different epithelial cell types and external stimuli shape this secretion pattern, we established and characterized a human intestinal organoid-on-chip (OoC) model. OoCs were used to evaluate barrier integrity and cell type-dependent expression profiles by RNA-sequencing and microscopy. Secretome analysis revealed asymmetric bidirectional secretion and a cell type-dependent response to LPS, flagellin, gliadin, and cytokines. In summary, we have developed an OoC model using human intestinal cells, offering a cutting-edge platform to study barrier function under physiological and pathophysiological conditions. This versatile tool holds significant promise for advancing our understanding of epithelial cells in innate immunity and driving the development of personalized therapeutic strategies.

## Highlights

• Human intestinal organoid-on-chip model for barrier and secretion studies
• Adjustable crypt-like or multi-lineage epithelial cell-type composition
• Asymmetric, bidirectional cytokine secretion depends on epithelial cell type
• Secretion is sensitive to inflammatory stimuli, bacteria, and dietary antigens

## Graphical abstract

**Figure:**
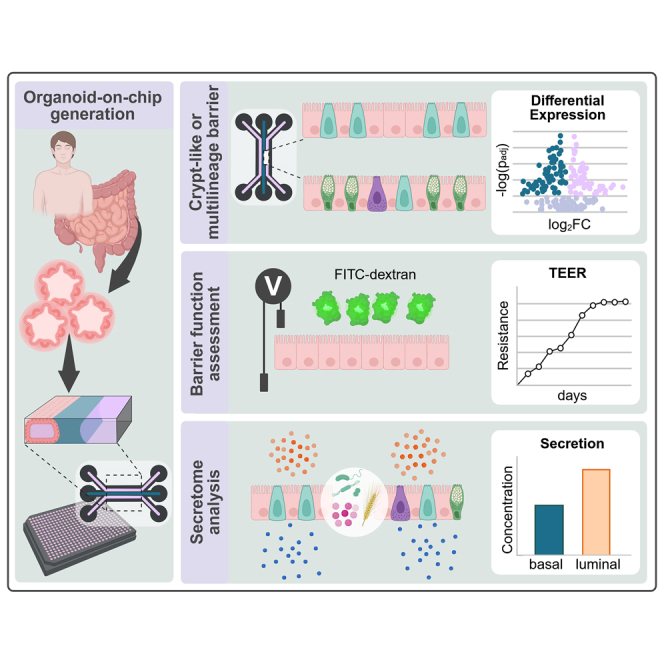

## Introduction

The intestinal epithelium comprises a heterogeneous cell population essential for absorption, secretion, and immune regulation. Epithelial cells, including colonocytes, goblet cells, enteroendocrine cells, and transit-amplifying (TA) cells form a barrier that separates luminal antigens from immune cells in the lamina propria and are continually replenished by crypt-residing stem cells. A mucus layer, enriched with antimicrobial peptides, prevents direct bacterial contact with epithelial cells while nourishing gut-residing bacteria. This close proximity between bacteria and immune cells needs to be further guarded to inhibit unwanted immune responses while inducing sufficient immune activation upon barrier damage. Therefore, it is essential that epithelial cells secrete cytokines and chemokines in an asymmetric manner with distinct luminal and basal levels. Thus, providing a safeguarding system that upon damage initiates a prompt immune response due to the rapid influx of cytokines and chemokines to the lamina propria.

Despite advancement in single-cell transcriptomics and spatial proteomics, our understanding of the specific factors secreted by epithelial cells remains limited. The emergence of human intestinal organoids as *in vitro* models has created new opportunities to study epithelial cell function in isolation free from confounding interference of immune cells or microbiota while resembling *in vivo* characteristics of the epithelial barrier. Further refinement of the culture medium now support the coexistence of proliferative cells and differentiated absorptive and secretory lineages, creating a multi-lineage (ML) model that better mimics *in vivo* intestinal epithelial barrier architecture.^1^ However, a major limitation of 3D organoid culture remains which is the limited simultaneous accessibility of the luminal and basal compartment making it impossible to study bidirectional cytokine release. Organoid-on-chip (OoC) models offer a promising solution by combining the biological relevance of organoids with the functional advantages of microfluidics. While initial studies relied on easily manageable cell lines to establish gut-on-chip models,^2^^,^^3^^,^^4^^,^^5^^,^^6^^,^^7^^,^^8^ great progress has been made to combine either human iPSC-derived,^9^^,^^10^^,^^11^ small,^3^^,^^12^^,^^13^ or large intestinal^14^^,^^15^^,^^16^^,^^17^^,^^18^^,^^19^ organoids with microfluidics. Various chip designs have been developed, including systems with porous membranes to separate apical and basal channels,^2^^,^^3^^,^^9^^,^^11^^,^^12^^,^^13^^,^^14^^,^^15^^,^^20^ many of which are commercially available but come at a high cost, and designs based on complex extracellular matrix (ECM) patterns, which require specialized equipment and expertise in microfabrication.^16^^,^^17^^,^^21^ In contrast, the here used membrane-free plate-based platform,^4^^,^^5^^,^^6^^,^^7^^,^^10^^,^^18^ stands out as the most accessible and cost-effective option currently available. It supports scalability, is compatible with state-of-the-art readouts, enables future integration of additional cell types, and offers a user-friendly format suitable for routine use in both research and preclinical applications.

The aim of this study was to combine insights from 3D organoid technology, identifying a medium that supports a more physiological epithelial barrier, with advancements in microfluidic technology to create a scalable platform. Thus, establishing a model that supports the analysis of spatial epithelial secretion profiles with regard to directionality and their dependencies on cell type diversity.

Highlighting the versatility of this system and its advantage over conventional organoids, we established an OoC for the evaluation of secretion profiles under steady-state and in inflammatory conditions, upon stimulation with bacterial components and food-borne antigens and uncover their dependencies on cell-type composition. Our model advances our understanding of human intestinal epithelial physiology and lays the groundwork for more physiologically relevant human models. By enabling higher-throughput experiments geared toward personalized medicine, it improves the translatability of research findings to clinical applications and supports the development of more effective therapies in intestinal diseases like IBD.

## Results

### Establishment of OoC using human intestinal organoids

**Figure 1:**
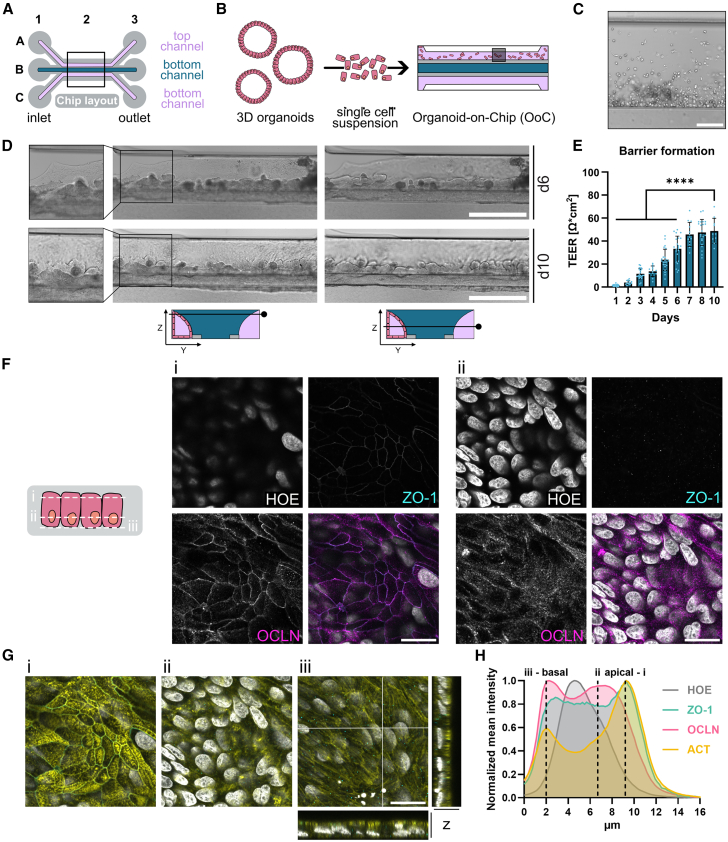
Barrier formation of human intestinal epithelial cells in OoC platform (A) Chip layout showing the membrane-free separation of the top and bottom channel by the ECM added to the gel channel. (B) Overview of single-cell dissociation from 3D organoids and cell seeding to the top channel of the OoC. (C) Dispersed cells 4 h after cell seeding. Scale bars, 100 μm. (D) Brightfield images of epithelial cells in OoC at the top (left) or middle (right) along the *z* axis on day 6 (top) and day 10 (bottom). The close-up shows the closing of the epithelial monolayer. Scheme shows the *z*-location of image acquisition. Scale bars, 500 μm. (E) Barrier formation shown as TEER build-up over the culture period of 10 days. Each dot represents one chip *n* = 13–23. Two donors in two independent experiments were analyzed. Data are shown as mean ± SD. One-way ANOVA with multiple comparisons was performed comparing all time points to values of day 10. Except days 7 and 8, all time points were significantly lower than on day 10. ∗∗∗∗ = <0.0001. (F) Confocal images showing the cell polarization with nuclei located toward the basal cell membrane (ii) and tight junctions toward the apical cell membrane (i). Nuclei (HOE) are shown in gray, ZO-1 in cyan, and occludin (OCLN) in magenta. Scale bars, 20 μm. (G) Confocal images showing the cell polarization with microvilli (i), intracellular space (ii), and stress fibers (iii) as well as the XZ and YZ projection. Nuclei are shown in gray, ZO1 in cyan, and actin in yellow. Scale bars, 20 μm. (H) Visualization of the mean intensity projection of staining for nuclei (HOE), ZO-1, occludin (OCLN), and actin (ACT) from the basal to the apical side in μm.

This study aimed to create a model system that closely replicates human intestinal epithelial barrier physiology while allowing spatial analysis of cytokine secretion. Traditional 3D organoids capture the complex epithelial cell types and donor-specificity found *in vivo* but lack easy access to the luminal space. Here, we combined human intestinal organoids with a microfluidics platform, enabling simultaneous access to the luminal and basal compartments while maintaining donor-specificity and enabling tuning of cell-type composition. Organoid lines from descending colon biopsies were cultured as a cell source for the OoC system, which uses commercially available chips designed without a membrane. Instead, a central gel channel with ECM separates the two cell culture compartments creating a more physiological cell-ECM interaction (Figure 1A). Adding single cells derived from organoids to the coated top channel initiated a 3D tubular structure formation over 10 days (Figure 1B), during which the dispersed cells proliferated into a continuous epithelial monolayer (Figures 1C and 1D). This was confirmed by a steady increase in transepithelial electrical resistance (TEER), which plateaued at 48.7 ± 11.1 Ω∗cm2 by day 10 (Figure 1E), indicating an established barrier. Immunofluorescence staining confirmed the presence of tight junction proteins ZO-1 and occludin (OCLN) at the intercellular spaces, regulating paracellular transport (Figure 1F). Additionally, actin staining showed microvilli development on the apical, lumen-facing side (Figure 1G), and further analysis confirmed proper cell polarization, with nuclei positioned basally and tight junctions and actin localized apically (Figures 1F–1H). Together, these results demonstrate that human intestinal organoids establish a functional epithelial barrier within the microfluidics platform separating the luminal and basal compartment.

### Functional epithelial barrier with bidirectional secretion pattern

**Figure 2:**
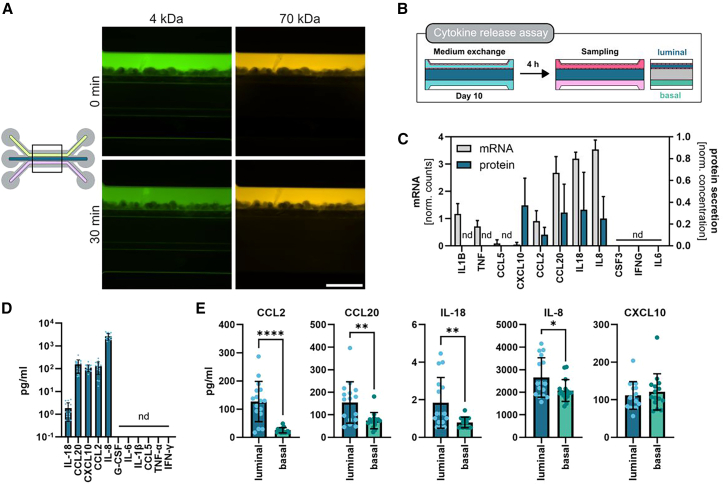
Directional cytokine release in human intestinal barrier model (A) Permeability assay on day 10 of culture shows successful barrier establishment. Both dextran molecules, 4 and 70 kDa, are retained in the luminal compartment after 30-min incubation. Scale bars, 500 μm. (B) Experimental design of cytokine assay. On day 10 of culture medium is exchanged, incubated for 4 h and then sampled luminally and basally. (C) Gene expression (gray) and cytokine abundance (blue) correlation shown as either normalized counts or min-max normalized concentration for each cytokine respectively of the 11-cytokine panel analyzed. Cytokine concentrations of the luminal compartment are shown. (D) Absolute values of measured cytokine concentration in the luminal compartment are shown. Cytokine concentrations that were below the detection limit are shown as not detectable (nd). (E) Graphs show the established asymmetric bidirectional cytokine release. Statistical significance was determined using *t* test and significance is represented as ∗ = 0.05, ∗∗ = 0.01, ∗∗∗ = 0.001, and ∗∗∗∗ = 0.0001. All measurements were performed on day 10 of culture in the CL condition. Gene expression data is shown for *n* = 6 and cytokine data for *n* = 16 of either 3 or 2 donors, respectively. All data are shown as mean with SD.

We next validated that the barrier was fully functional and capable to retain macromolecules as suggested by the TEER measurements. Permeability assay using 4 and 70 kDa dextran fluorescently labeled were not able to pass the epithelial barrier confirming barrier integrity (Figure 2A). To explore the bidirectional secretion pattern of epithelial cells under steady-state condition (Figure 2B), we performed a multiplex ELISA targeting cytokines and chemokines. Our analysis revealed robust secretion of CCL2 (MCP-1), CCL20, IL-18, IL-8 (CXCL8), and CXCL10 while IL-6 and G-CSF (CSF3) remained below the detection limit (Figure 2C). This secretion pattern was largely reflected at the transcription level, except for CXCL10 with a relatively low expression level indicating potential post-transcriptional regulation. IL-8 was identified as the most abundant cytokine on the luminal side, with concentrations reaching 2653 ± 876 pg/mL, while IL-18 was present at the lowest levels, at 1.84 ± 1.35 pg/mL. Intermediate concentrations were observed for CCL2, CCL20, and CXCL10 at 127.2 ± 74.5 pg/mL, 154.6 ± 92.1 pg/mL, and 111.4 ± 36.7 pg/mL, respectively (Figure 2D). The high levels of IL-8 and CCL2 are consistent with previous studies using a transwell system, additionally we could identify the secretion of CCL20, IL-18 and CXCL10 and show the absence of IL-6 and G-CSF (CSF3) under steady-state condition.

An asymmetric secretion pattern was observed for all detected cytokines and chemokines, with the exception of CXCL10. Luminal concentrations of CCL2 were 5.1 times higher than those in the basal compartment, and similar directionality was seen with CCL20, IL-18, and IL-8, showing 2.1-fold, 2.3-fold, and 1.2-fold higher concentrations, respectively (Figure 2E). These results demonstrate that our intestinal epithelial barrier model not only enables precise analysis of bidirectional cytokine secretion but also captures a key epithelial function which is the formation of directional concentration gradients across the barrier. This highlights the active role of the epithelium in maintaining intestinal homeostasis and communicating with its environment as another layer of defense.

### ML OoC with distinct transcriptomic profile

**Figure 3:**
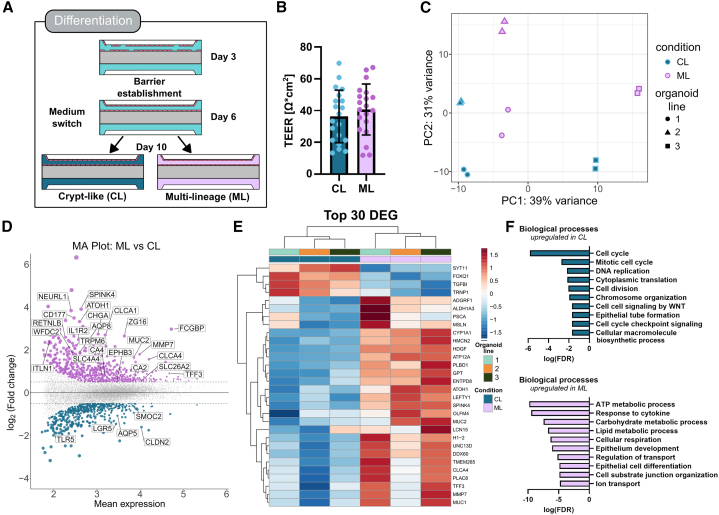
Differentiation of epithelial cells in OoC (A) Experimental setup showing the application of two different culture media starting on day 6. (B) Barrier integrity on day 10 shown as TEER. Each dot represents one chip, *n* = 20. Two donors in two independent experiments were analyzed. Data are shown as mean ± SD. Indifference was determined by unpaired *t* test. (C) Scatterplot of the first two principal components (PC1 and PC2) showing clustering of organoid lines (shapes) and differentiation state (color). (D) MA plot representing differentially up- and downregulated genes (*p*_adj_ < 0.05) comparing ML to CL condition. Several genes of interest are labeled. (E) Heatmap representing the top 30 differently expressed genes. (F) GO enrichment analysis (biological processes) comparing ML and CL condition. Selected terms of the top 70 are shown. Scores of GO terms indicating the enrichment FDR values.

Next, we explored the possibility of inducing ML differentiation while maintaining self-renewal capacity as shown for conventional 3D organoid culture.^1^ This approach supports the simultaneous presence of differentiated cell types of both the secretory and absorptive lineage alongside a proliferative cell population, mimicking the heterogeneous cell-type composition of the native intestinal epithelium. We compared the widely used, conventional medium^22^ composition which mostly maintains the crypt-like (CL), proliferative population to a refined medium composition reported to support cellular diversity generating a ML phenotype (Figure 3A).^1^^,^^23^ To assess barrier integrity, we measured transepithelial electrical resistance on day 10 of culture and detected no differences between ML and CL condition, 40.7 ± 16.0 Ω∗cm^2^ and 36.3 ± 16.6 Ω∗cm^2^ respectively, indicating a balance between shed cells, differentiation and proliferation in ML condition (Figure 3B). Next, we wanted to understand how the transcriptomic profiles of both conditions changed analyzing three organoid lines with two technical replicates each. Principal component analysis (PCA) of the RNA-seq data showed a distinctive expression profile which was dependent on cellular diversity but also showed organoid line-specific clustering (Figures 3C and S1). In the downstream analysis, we accounted for organoid line variability, thus focusing on effects induced by the ML condition. In total, 2,771 differentially expressed genes (*p*_adj_ < 0.05) were identified (Figure 3D). Interestingly, several goblet cell marker genes (MUC2, ATOH1, SPINK4, CLCA4, and TFF3) were among the top 30 differentially expressed genes (Figure 3E) indicating the emergence of the secretory lineage. Gene ontology (GO) enrichment analysis was performed to identify affected underlying pathways. The analysis uncovered gene sets involved in biological processes like cell cycle, cytoplasmic translation, DNA replication, and epithelial tube formation enriched in CL condition indicating a proliferation dominated state. In contrast, the ML condition was enriched for gene sets connected to metabolic processes, cellular respiration, epithelial cell differentiation, and ion transport indicating the presence of more specialized cell types (Figure 3F). These results show the successful generation of OoCs with an intact barrier and heterogeneous cell-type composition indicating the ability to study physiological relevant, ML epithelial functions.

### Co-existence of mucus producing and proliferative cells

**Figure 4:**
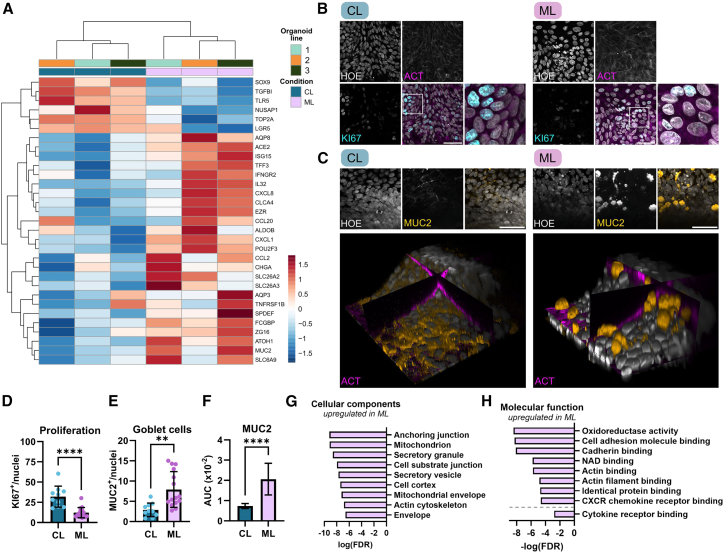
Distinct cell-type composition of *in vitro* intestinal epithelial barrier (A) Heatmap showing selected cell-type markers detected as DEG. See also Figure S1. (B) Confocal images showing staining for proliferative marker KI67 (cyan) along with staining for nuclei (HOE, gray) and actin (ACT, magenta). Scale bars, 50 μm. (C) Confocal images showing staining for goblet cells maker MUC2 (yellow) along with staining for nuclei (HOE, gray) and actin (ACT, magenta). Lower panels show 3D reconstruction with ortho-slices. Scale bars, 50 μm. (D) Quantification of KI67^+^ cells normalized to total cell number. See also Figure S2. (E) Quantification of MUC2^+^ cells normalized to total cell number. See also Figure S2. (F) Quantification of the mean intensity of MUC2 normalized to total cell number and analyzed as area under the curve (AUC) for statistical analysis. See also Figure S2. (G) GO enrichment analysis (cellular components) comparing ML to CL condition. (H) GO enrichment analysis (molecular function) comparing ML to CL condition. For (D)–(F), each dot represents one chip *n* = 10–15. For (D)–(G), 2–3 donors in two independent experiments were analyzed. Data are shown as mean ± SD. Statistical significance was determined using *t* test and significance is represented as ∗ = 0.05, ∗∗ = 0.01, ∗∗∗ = 0.001, and ∗∗∗∗ = 0.0001. In (G) and (H), scores of GO terms indicate the enrichment FDR values.

To further characterize the cell diversity in the two conditions, cell-type-specific markers were analyzed. *In vivo* intestinal stem cells give rise to TA cells that differentiate into either the absorptive lineage, colonocytes, or the secretory lineage consisting of goblet and enteroendocrine cells and rather rare Tuft and M cells.^1^^,^^23^^,^^24^ The comparison between ML and CL condition, showed a decreased expression of stem cell markers LGR5 and SOX9 in ML condition which was in line with data of 3D organoids and was accompanied by a reduction of TA cell markers TOP2A and NUSAP1 (Figure 4A).^1^ In contrast, many markers for differentiated cells were upregulated amongst them markers for colonocytes like ALDOB and EZR. Additionally, ion transporters, such as CA1, SLC6A9, SLC26A2, and SLC26A3, were upregulated, which are crucial for pH balance and salt uptake, along with AQP3 and AQP8, key aquaporins for water absorption.^25^ Given that the primary role of the colon involves the absorption of salt and water, this upregulation suggests that in ML condition the colonic absorptive function is preserved. Furthermore, an increase in the expression of markers associated with non-canonical colonocytes was observed indicated by the presence of IL32, CXCL8, and ISG15 (Figure 4A).^23^ The observed increased expression of CHGA and POU2F3 in ML suggested the presence of EECs and Tuft cells, respectively. However, secretory lineage markers were dominated by an upregulation of goblet cell-specific genes, among them mucus components MUC2, TFF3, and CLCA4 as well as several markers for mature goblet cell ITLN1, SPDEF, FCGBP, and ZG16 (Figure 3D). We also detected the expression of MUC5B, expressed by crypt-base goblet cells along with membrane-bound mucins MUC1 and MUC13, essential for mucus production *in vivo* (Figure 3E and Table S2).^24^^,^^26^ This increase in secretory lineage markers is in line with scRNA-seq data from 3D organoids and complex ECM-patterned OoC.^1^^,^^16^ Overall, we show the emergence of the secretory and absorptive lineage in ML condition compared to the CL condition which in contrast is dominated by proliferative cells.

To confirm these results on protein level, we chose two markers which represent the major characteristic of either the CL or ML condition: KI67, a marker for proliferation and MUC2, a marker for mucus secretion by goblet cells. Nuclei which were positive for KI67 were present in both conditions but appeared with higher intensity in the CL condition (Figures 4B and S2A). Further analysis revealed a significantly higher number of KI67^+^ cells in CL condition (Figure 4D) confirming its higher proliferative capacity while showing the preserved proliferative potential in ML condition. Typical mucin granules accumulating toward the apical cellular membrane, as characteristic of goblet cells, were only detectable in ML condition as shown in the 3D image reconstruction (Figure 4C). Additional analysis revealed a significant increase of goblet cell number (Figure 4E) and higher MUC2 abundance across the total cell height (Figures 4F and S2C) confirming the presence of secretory granules in ML as suggested by the GO enrichment analysis (Figure 4G). Taken together, these findings imply that the CL condition predominantly features proliferating cells, as evidenced by biological processes related to cell division and the detection of high numbers of KI67^+^ cells. In contrast, the ML condition appears to be predominated by differentiated cells, as suggested by biological processes associated with ion transport and secretion, alongside the identification of goblet cells labeled by MUC2 while maintaining proliferative potential similar to the barrier *in vivo*.

### Increased secretion of IL-8, CCL2, and CCL20 correlates with emergence of differentiated cell

**Figure 5:**
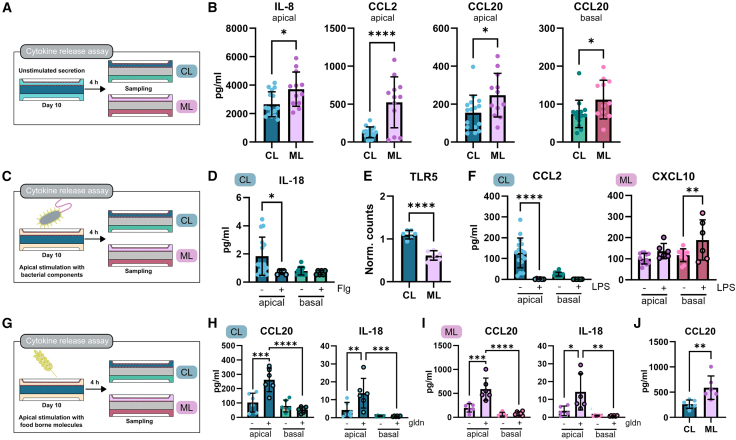
Cell-type-dependent cytokine release under basal and stimulated conditions (A) Experimental design of cytokine assay under steady-state condition. (B) Cytokine concentrations in the luminal and basal compartment of unstimulated CL and ML condition are shown as indicated. (C) Experimental design of cytokine assay when stimulated with bacterial components flagellin and LPS. (D) IL-18 concentrations in luminal and basal compartment of CL condition after flagellin (+Flg) stimulation and the respective controls (−) are shown. (E) Gene expression of TLR5, identified as differentially expressed gene in RNA-seq analysis, is shown as normalized counts. Data represent 3 donors in *n* = 2 replicates. (F) CCL2 and CXCL10 concentrations in luminal and basal compartment of CL and ML condition, respectively, after LPS stimulation (+) and the respective controls (−) are shown. (G) Experimental design of cytokine assay when stimulated with gliadin, a food-borne molecule. (H) CCL20 and IL-18 concentrations in luminal and basal compartment in CL condition after gliadin (+gldn) stimulation and the respective digestion controls (−) are shown. (I) CCL20 and IL-18 concentrations in luminal and basal compartment in ML condition after gliadin (+gldn) stimulation and the respective digestion controls (−) are shown. (J) CCL20 concentrations in the luminal and basal compartment of unstimulated CL and ML condition are shown. All measurements were performed on day 10 of culture after 4 h stimulation. Cytokine data is shown for 2 donors, *n* = 12–16 for controls and *n* = 6 for stimulation and digestion controls. All data are shown as mean with SD. Statistical significance was determined using (B), (J) unpaired *t* test, (E) paired *t* test, and (D), (F), (H), and (I) one-way ANOVA with Šídák’s multiple comparison. Significance is represented as ∗ = 0.05, ∗∗ = 0.01, ∗∗∗ = 0.001 and ∗∗∗∗ = 0.0001. See also Figure S3.

Considering that the CL and ML condition showed a distinct cell-type composition and GO enrichment analysis showed changes in gene sets connected to chemokine and cytokine receptor binding (Figure 4H), we analyzed the cytokine secretion profile. Thus, the supernatant of CL and ML condition was sampled after 4 h without stimulation to assess differences under steady-state conditions (Figure 5A). The analysis showed that in both conditions a similar cytokine composition was secreted with a preserved luminally dominated directionality (Figures 2D and S3A). Detailed analysis revealed that with increased cellular diversity a significantly higher secretion of IL-8, CCL2, and CCL20 was observed while IL-18 and CXCL10 levels remained unaltered (Figure S3B). Luminal IL-8 and CCL2 concentrations were 1.4- and 4.2-fold higher, respectively, while basal levels remained unchanged (Figure S4C). CCL20 secretion was higher in both compartments with 1.6- and 1.5-fold higher luminal and basal levels, respectively. The results on protein level were in line with our RNA-seq data identifying IL-8 (CXCL8), CCL2, and CCL20 as significantly upregulated genes (Figure 4A). Collectively, these results show the overall impact of cellular diversity on cytokine release suggesting a crypt-villus dependent secretion pattern in native epithelium.

### Response to bacterial stimuli is directed and depends on epithelial cell-type composition

We next tested the functionality of the epithelial barrier in response to bacterial components, dietary antigens and cytokines and analyzing their effect on the cytokine secretion profile. First, we exposed the epithelial cells luminally to flagellin and LPS mimicking the luminal presence of bacteria *in vivo* (Figure 5C). We analyzed the cytokine composition of the supernatant after 4-h incubation to determine whether epithelial cells in our model could sense bacterial components via TLR4 and TLR5, both of which are expressed. Upon flagellin stimulation, a TLR5 ligand, we observed a luminal decrease in IL-18 secretion, specifically in the CL condition (Figures 5D and S3E). This response aligns with a reduction in TLR5 expression in ML condition (Figures 5E and 4A), suggesting a diminished sensitivity to TLR5 stimulation in more differentiated epithelial cells.^27^ The analysis of the response to LPS showed a different cytokine secretion pattern with a decrease of luminal CCL2 release (5.1-fold) which fell below the detection limit in CL condition (Figures 5F, S3E, and S3F). In the ML condition, we detected a 1.6-fold increase of basal CXCL10 secretion (Figure 5F). Both changes were highly specific for the given cell-type composition. Analyzing the expression of TLR4, essential for LPS sensing, revealed no differences between CL and ML condition suggesting a TLR4-independent cell-type dependent regulation (Figure S3G).^28^ In summary, the analysis demonstrates that bacterial stimuli induce cell-type-specific responses with the capability of distinguishing between TLR4- and TLR5-dependent stimuli even upon short-term exposure.

### Gliadin induces IL-18 and CCL20 secretion

As a next step, we were interested in testing if we would be able to detect epithelial cell responses to food-borne molecules. We chose gliadin since it is the immunogenic component of gluten which can trigger celiac disease.^29^ Even though epithelial cells are the first responders to any food-borne molecule their cytokine response to gliadin is so far unknown. The analysis of the secretion profile after luminal gliadin exposure (Figure 5G) revealed increased IL-18 and CCL20 levels into the luminal compartment (Figures 5H and 5I) altering the asymmetric cytokine levels. IL-18 secretion increased 3.2- and 1.7-fold while CCL20 secretion increased 2.5- and 3.0-fold in CL and ML condition, respectively. Comparing both conditions showed a significantly higher luminal CCL20 concentration in ML (Figure 5J) suggesting a higher responsiveness to gliadin in differentiated cells. This could be further explained by the increased expression of CXCR3, targeted by gliadin, in ML condition (Figure S2H). Overall, our data show that gliadin exposure leads to an epithelial cell response with an altered cytokine profile which potentially alters immune cell recruitment and activation *in vivo*.

### G-CSF dominates the response to an inflammatory condition

**Figure 6:**
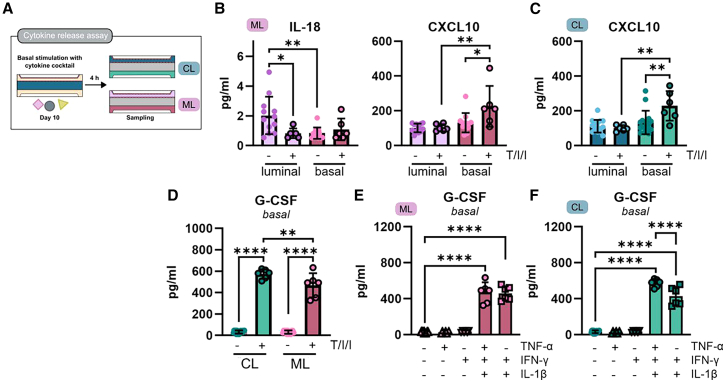
Cytokine release upon stimulation with proinflammatory cocktail (A) Experimental design of cytokine assay when stimulated with proinflammatory cocktail. (B) IL-18 concentrations in luminal and basal compartment of ML condition after stimulation with TNF-α (T), IL-1β (I), and IFN-γ (I) and the respective controls (−) are shown. (C) CXCL10 concentrations in luminal and basal compartment of CL condition after stimulation with TNF-α (T), IL-1β (I), and IFN-γ (I) and the respective controls (−) are shown. (D) G-CSF concentrations in basal compartment of CL and ML condition after stimulation with TNF-α (T), IL-1β (I), and IFN-γ (I) and the respective controls (−) are shown. (E) Basal G-CSF concentrations in ML condition after indicated stimulations and the respective controls (−) are shown. (F) Basal G-CSF concentrations in CL condition after indicated stimulations and the respective controls (−) are shown. All measurements were performed on day 10 of culture after 4 h stimulation. Cytokine data are shown for 2 donors, *n* = 10 for controls and *n* = 6 for stimulation. All data are shown as mean with SD. Statistical significance was determined one-way ANOVA with Šídák’s multiple comparison. Significance is represented as ∗ = 0.05, ∗∗ = 0.01, ∗∗∗ = 0.001, and ∗∗∗∗ = 0.0001. See also Figure S4.

Finally, we wanted to investigate how the secretion profile of our OoC would change during intestinal inflammation. To mimic an inflammatory state typical for IBD, we exposed the epithelial cells basally to TNF-α/IL-1β/IFN-γ as it would occur *in vivo* from immune cells residing in the lamina propria (Figure 6A). Interestingly, analysis of the supernatant revealed a decrease (2.4-fold) of luminal IL-18 secretion exclusive to the ML condition (Figures 6B and S4A) abolishing asymmetric levels observed under steady-state conditions. In contrast, basal CXCL10 secretion was increased by 1.7-fold in both conditions inducing asymmetric levels. The most prominent change, however, was the detection of G-CSF in the basal compartment which was undetectable under steady-state. Upon stimulation with the proinflammatory cytokine cocktail, its secretion increased significantly by 17.1- and 14.7-fold in CL and ML condition, respectively (Figures 6D and S4B). The response in the CL condition was significantly higher than in ML condition (Figure 6D). Stimulation with either TNF-α or IFN-γ alone was not able to induce G-CSF secretion (Figures 6E and 6F). In contrast, IL-1β was able to induce G-CSF secretion at similar levels as TNF-α/IL-1β/IFN-γ in ML condition. In the CL condition, stimulation with TNF-α/IL-1β/IFN-γ showed an additive effect with a significantly higher G-CSF secretion than IL-1β stimulation alone. Taken together, we could identify G-CSF as a so far understudied cytokine released by epithelial cells upon IL-1β stimulation.

Taken together, we show that that our OoC model is able to detect asymmetric bidirectional secretion patterns which depend on cell-type diversity and stimulation. It highlights the important role epithelial cells play as communicator and regulator of immune cell recruitment and activation under physiological conditions and during inflammatory conditions like IBD.

## Discussion

Here, we report the development of human colon organoids-on-chip that overcome a key limitation of conventional 3D organoid culture, the inability to access both luminal and basal compartments simultaneously, while preserving essential organoid features like self-organization, proliferation, and differentiation potential as well as patient specificity. Additionally, we increase physiological resemblance by introducing shear stress and increase through-put. Compared with other bioengineered organoid models,^12^^,^^14^^,^^20^ our OoC features a near-physiological environment with direct ECM-cell interaction and maintenance of a 3D structure by the formation of a tubular, gut tube-like structure. Building on previous work,^1^^,^^4^^,^^7^^,^^13^^,^^18^ we integrate a commercially available microfluidics platform with 3D human intestinal organoids to establish a robust, reproducible, ML epithelial model with improved cell diversity. We characterize the cell-type composition of the OoC using the commonly used medium supporting the proliferative compartment and show that with refined conditions cell-type diversity can be improved. While other bioengineering approaches might be able to resemble *in vivo* spatial crypt-villus architecture with greater accuracy, they suffer from low throughput and fabrication complexity.^16^ Our approach demonstrates that it is possible to increase throughput while also improving cell diversity,^6^^,^^10^^,^^18^ advancing the application of OoC technology for translational research and personalized medicine.

Our findings show that the organoids derived from descending colon form a functional epithelial barrier resembling native tissue. The separation of the luminal from basal compartment was created by proper cell polarization with the accumulation of actin and tight junction proteins toward the apical membrane.^30^^,^^31^^,^^32^ In contrast to cell lines reporting up to 10-fold higher values,^6^^,^^8^ TEER values of our model mimicked values reported for *ex vivo* human epithelial colon tissue ranging from 30 to 40 Ω∗cm^2^.^33^^,^^34^ This validated our OoC model to be suitable for analyzing bidirectional secretion and providing a tool for spatially defined stimulation which is experimentally difficult to accomplish in conventional 3D organoid culture.

Another important finding is that we could improve the cell diversity in the OoC by using refined culture conditions (ML) instead of the commonly used medium composition (CL).^1^^,^^35^ Bulk RNA-seq and immunofluorescence analysis showed the donor-dependent differences between the CL and ML condition and showed the shift from a CL, proliferation-dominated phenotype to a ML phenotype with maintained self-renewing capacity. In contrast to other reports analyzing OoCs,^14^^,^^18^ we were not able to detect goblet cells in the CL condition which is in line with reports of 3D organoids^1^ and the suppressive role on the secretory line by p38 mitogen-activated protein kinase (MAPK) inhibitor contained in CL medium. In ML condition, we detected an increase of markers associated with ion transport and secretion as well as changes in genes connected to tight junctions and antimicrobial defense. The enhanced cell diversity of the ML condition reflects the native tissue complexity with proliferative, absorptive and secretory compartments better than the CL condition providing a near-physiological platform to functionally analyze colon-specific epithelial physiology and cell responses to various stimuli.

We hypothesized that cytokine secretion by epithelial cells is asymmetrical and cell-type-dependent. Since we were interested in understanding primary effects on secretion, rather than the integrated response caused by gene expression changes and receptor engagement by endogenously secreted factors, we exposed the epithelial cells only for 4 h to the specific stimulus. Our data revealed the secretion of CCL2, CCL20, CXCL10, IL-18, and IL-8 to both the luminal and basal compartment. Consistent with recent reports IL-8 was the most abundant measured cytokine.^36^^,^^37^ While CXCL10 was detected at similar levels in both compartments, CCL2, CCL20, IL-8, and IL-18 were secreted asymmetrically, showing lower levels in the basal compared to the luminal compartment which is in contrast to reports using Caco cells or iPSCs.^6^^,^^7^^,^^10^ This might be caused by their limited ability to represent the physiological cellular complexity and cancerous origin or in case of iPSCs, their different state of maturity. We could show that enhanced cell diversity increased luminal secretion of CCL2, IL-8, and CCL20, with the latter also showing elevated basal secretion. This followed the same pattern as seen on mRNA level with a significantly upregulation of CCL20 and IL-8 (CXCL8) expression as well as enrichment of chemokine- and cytokine-related pathways in ML condition. These findings are in line with recent scRNA-seq data of human colon organoids showing increased CXCL8 expression in differentiated cells.^23^ In conclusion, our data provides insights to a cell-type-dependent secretion pattern that allows for a spatially defined response *in vivo* and highlights the relevance of cell heterogeneity in *in vitro* models. The greater luminal cytokine abundance might serve as an early warning system which upon barrier damage leads to a sudden increase of cytokine levels at the basal side attracting immune cells residing in the lamina propria to the site of injury.

As a next step, we focused on testing whether our system could resolve the stimulation-specific response and show differences depending on cell diversity. Our data suggests that the proliferative compartment is more sensitive to TLR5 stimulation than a heterogeneous epithelial barrier and similarly TLR4 engagement leads to cell-type-specific responses. Of note, in contrast to the report by Kayisoglu et al.,^38^ we could not detect IL-8 secretion upon stimulation with flagellin. This could be explained by the relatively short stimulation duration of only 4 h. Similarly, we were not able to detect increased IL-8 upon stimulation with LPS as shown by Wang et al.^37^ which might be due to our much lower LPS concentration (100 ng/mL compared to 5 μg/mL^37^). Our results indicate that responses to bacterial antigens are spatially organized along the crypt axis, highly sensitive to concentration and exposure time. *In vivo*, the crypt is shielded by a mucus layer, and bacterial translocation into the proliferative compartment may only occur following a disruption of this protective barrier. Thus, it is expected that cells of the proliferative compartment which are protected by the crypt architecture *in vivo* mount a different response to stimuli than differentiated cells located closer toward the surface and with higher chances for encountering host-microbe interaction. Gliadin, the immunogenic component of gluten, increased luminal secretion of CCL20 and IL-18 indicating an induced change in the chemotactic gradient. Upon barrier compromise as seen in celiac disease and IBD, CCL20 and IL-18 might be able to drive immune cell migration to the lamina propria which might explain the reported beneficial effect of an gluten-free diet^39^^,^^40^^,^^41^^,^^42^ and increased serum CCL20 and IL-18 levels in celiac patients.^43^^,^^44^ The exposure to a proinflammatory cytokine mix (TNF-α, IL-1β, and IFN-γ), typical for modeling IBD *in vitro*, induced a loss of the IL-18 gradient and induction of basal CXCL10 release with the latter being similar to other reports.^7^^,^^45^ Interestingly, the most pronounced effect was the induction of basal G-CSF secretion which was undetectable under steady-state conditions. Additional analysis of the stimulation with each individual cytokine showed the dependency on IL-1β which is in contrast to data from transwell studies using ileal organoids which detected G-CSF induction upon TNF-α stimulation.^45^ G-CSF is an important modulator of neutrophil immunity and was shown to have anti-inflammatory effects by the modulation of T cell activation and function.^46^^,^^47^^,^^48^ Since it plays a protective role in DSS colitis further studies are of high interest to understand how G-CSF release can be modulated to find alternative treatment options for IBD.^49^ Overall, our results highlight the cell-type dependent change in secretion pattern as response to external stimuli.

Our data highlight the potential of the established OoC model in investigating the largely unexplored secretion patterns of epithelial cells and their dependencies on cell-type composition and stimuli, a critical area for advancing our understanding of intestinal physiology and pathophysiology. This physiologically relevant platform enables diverse stimulations, compound testing, and the integration of additional cell types, representing an important step toward a human-relevant, personalized prediction system that bridges the gap between preclinical research and clinical application. Our OoC model provides a validated, physiologically relevant framework for investigating intestinal secretion dynamics and advancing patient-centered, targeted therapeutic development.

### Limitations of the study

The OoC model presented here features a membrane-free design for epithelial barrier generation, offering a key advantage over conventional systems. However, this design also imposes a limitation: culturing beyond 14 days requires modifications to the ECM composition. This could be accomplished by cross-linking the matrix as this was shown to improve matrix strength.^50^ Additionally, the platform is restricted to a planar structure, differing from the complex crypt-villus architecture found *in vivo*. While our OoC incorporates multiple cell types and allows differentiation to be modulated through medium composition, a more advanced design is necessary to study morphogen gradients and the 3D organization of the epithelial barrier as shown elsewhere.^16^^,^^21^

Finally, although this OoC setup enables higher throughput, one plate consists of 40 chips, compared to traditional cultures, it also comes with the drawback of smaller cell numbers and limited sample volumes. Consequently, downstream analyses are more challenging due to the lower yield of RNA and protein. This limitation can be addressed using specialized methods optimized for low-yield samples or by pooling material from multiple OoCs to increase sample quantities.

## Resource availability

### Lead contact

For additional details or resource requests, please contact the lead author, Martín Resnik-Docampo (resnik@bio.mx).

### Materials availability

Organoid lines generated fall under the restrictions given by our MTA with Novobiosis and cannot made available.

### Data and code availability

• RNA sequencing data have been deposited at DDBJ (DDBJ: DRA023605) and are publicly available as of the date of publication.
• Other data and images that support the findings of this study are available on request from the lead contact.
• This paper does not report original code.
• Any additional information required to reanalyze the data reported in this paper is available from the lead contact upon request.

## Acknowledgments

We would like to thank Hugo de Jonge for providing the R-Spondin 1 cell line; Stephanie Münchau for helpful discussion and experimental support; Christoph Becker and Daigen Xu for valuable input and guidance. We also thank Novogene for their assistance in RNA sequencing and the Nikon Imaging Center at the BioQuant, 10.13039/501100001661University of Heidelberg for providing access to their imaging facility. Special recognition goes to the patients who donated their intestines for research. The research was funded by 10.13039/100009945Merck KGaA, Darmstadt, Germany during the employment of MRD, IVH, SE, and MS at the BioMed X Institute. This research has received funding from the 10.13039/501100001659Deutsche Forschungsgemeinschaft (10.13039/501100001659DFG [10.13039/501100001659German Research Foundation]) – SFB/TRR369 DIONE – 501752319 (A02). Further support was provided by the Interdisciplinary Center for Clinical Research of the 10.13039/501100001652Friedrich-Alexander-Universität Erlangen-Nürnberg (Pilot project funding P175, Junior project funding J117, Synergery Grant S4). The graphical abstract was created in BioRender. Hensel, I. (2025), https://BioRender.com/7pfvezy.

## Author contributions

Conceptualization, I.V.H. and M.R.-D.; formal analysis and visualization, I.V.H. and S.E.; methodology, validation, and writing – original draft, I.V.H.; investigation, I.V.H. and M.S.; writing – review and editing, I.V.H., C.G. and M.R.-D.; resources, M.R.-D.; funding acquisition, C.G. and M.R.-D.; project administration, and supervision, M.R.-D.

## Declaration of interests

The authors declare no competing interests.

## STAR★Methods

### Key resources table

**Table:** 

REAGENT or RESOURCE	SOURCE	IDENTIFIER
**Antibodies**		

Alexa Fluor 488 Goat anti-Rabbit IgG (H + L)	Life Technologies	A11034; RRID: AB_2576217
Alexa Fluor 568 Goat anti-Mouse IgG (H + L)	Life Technologies	A11031; RRID:AB_144695
KI-67 Polyclonal Antibody	Abcam	ab15580; RRID:AB_443209
Mucin-2 Monoclonal Antibody	Santa Cruz Biotechnology	sc-515032; RRID:AB_2815005
Occludin Monoclonal Antibody	Life Technologies	33–1500; RRID:AB_2533101
ZO-1 Polyclonal Antibody	Life Technologies	61–7300; RRID:AB_2533938

**Biological samples**		

Human descending colon organoids	Novobiosis	–

**Chemicals, peptides, and recombinant proteins**		

1 M HEPES Buffer Solution	Capricorn Scientific	HEP-B
A83-01	Sigma Aldrich	SML0788
advanced DMEM/F12	Thermo Fisher	12634028
B27	Thermo Fisher	17504044
Collagen IV derived from human placenta	Sigma-Aldrich	C5533
Dulbecco’s PBS (1x), w/o Ca & Mg, w/o Phenol Red	Capricorn Scientific	PBS-1A
EGF	Peprotech	315–09
FGF-2	Peprotech	100-18C
Fluorescein isothiocyanate–dextran 3000–5000 Da (FITC 4 kDa)	Sigma-Aldrich	FD4
Gliadin from wheat	Sigma Aldrich	G3375
GlutaMAX	Thermo Fisher	35050038
HBSS w/o Ca, Mg	Fisher Scientific	14175129
HEPES	Capricorn Scientific	HEP-B
Hoechst 33342	Fisher Scientific	H3570
Hydrochloric acid	Sigma Aldrich	H9892
IFN-γ	Peprotech	300–02
IGF-1	BioLegend	590906
IL-1β	Peprotech	200-01B
Leu[15]-Gastrin I	Sigma Aldrich	G9145
Lipopolysaccharides from Escherichia coli O55:B5	Sigma Aldrich	L4524
Matrigel	Corning	356231
N-Acetyl-Cysteine	Sigma Aldrich	A7250
Nicotinamide	Sigma Aldrich	A7250
Noggin	Peprotech	250–38
Paraformaldehyde solution 4% in PBS (4% PFA)	Santa Cruz Biotechnology	sc-281692
Pepsin from porcine gastric mucosa	Sigma Aldrich	P6887
Phalloidin ATTO 643	ATTO-TEC	AD 643-81
Primocin	InvivoGen	ant-pm-1
Recombinant Flagellin from S. typhimurium	Invitrogen	tlrl-epstfla
SB202190	Tocris	1264
Sodium hydroxide solution	Sigma Aldrich	72068
StemPro Accutase Cell Dissociation Reagent	Gibco	A11105
surrogate Wnt	U-protein	*N*-001
TeloCol®-6 Bovine Type I Collagen Solution, 6 mg/mL	Advanced Biomatrix	5225
Tetramethylrhodamine isothiocyanate–Dextran 65000–80000 Da (TRITC 70 kDa)	Sigma-Aldrich	T1162
TNF-α	Peprotech	300-01A
Trypsin from bovine pancreas	Sigma Aldrich	T9201
Ultrapure 0.5 M EDTA Solution	Thermo Fisher	15575020
Y-27632	Hölzel Diagnostika	HY-10583

**Critical commercial assays**		

LEGENDplex Human Inflammation Panel 1	BioLegend	740809
RNeasy Plus Micro Kit	Qiagen	74034

**Deposited data**		

RNA sequencing data	DDBJ	DRA023605
Experimental models: Cell line		
R-Spondin1-conditioned medium (stably transfected HEK293T cells)	kindly provided by Hugo de Jonge	–

**Software and algorithms**		

Fiji Software	Schindelin et al.^51^	https://imagej.net/software/fiji/#downloads
BioVOXXEL plugin	Brocher et al.^52^	biovoxxel.de
DESeq2	Love et al.^53^	https://bioconductor.org/packages/devel/bioc/html/DESeq2.html
hypeR package	Federico et al.^54^	https://www.bioconductor.org/packages/release/bioc/html/hypeR.html
Molecular Signatures Database (MSigDB)	Liberzon et al. and Dolgalev et al.^55^^,^^56^	https://www.gsea-msigdb.org/gsea/msigdb
nf-core/rnaseq pipeline (version 3.8.1)	Patel et al.^57^	https://nf-co.re/rnaseq/3.14.0/
Salmon	Patro et al.^58^	https://github.com/COMBINE-lab/salmon
STAR alignment	Dobin et al.^59^	https://github.com/alexdobin/STAR
StarDist	Schmidt et al.^60^	https://stardist.net/

**Other**		

1.5 mL tubes	Sarstedt	72.706
15 mL conical tubes	neolab Migge	352070
24-well plate	Corning	3526
40 μm EASYstrainer Cell Sieves	Greiner Bio-one	542140
96-well plate	neolab Migge	C-8206
Adhesive Plate Seals	Fisher Scientific	AB0558
Combitips Advanced™ Biopur™ Pipette Tips, 0.1 mL	Eppendorf	30089650
Combitips Advanced™ Biopur™ Pipette Tips, 2.5 mL	Eppendorf	12614597
Confocal microscope (e.g., AX)	Nikon	–
Costar Flat Bottom 24-well Cell Culture Plates	Corning	3526
Electronic Multi-Dispenser Pipette	Eppendorf	4987000380
Fluorescence microscope and brightfield microscope (e.g., Eclipse Ti2)	Nikon	–
Microscope stage incubator	Tokai Hit	–
OrganoFlow	Mimetas	–
OrganoPlate 3-lane 40	Mimetas	–
OrganoPlate stand	Mimetas	–
OrganoTEER	Mimetas	–
Pipette controller accu-jet pro	Brand	10234982
Pipette tip, 200 μL, Biosphere plus sterile	Sarstedt	70.760.202
Pipette, 10 mL	Greiner Bio-one GmbH	607180
Syringe filter, PVDF, 0.22 μm	Roth	P666.1

### Experimental model and study participant details

#### Human intestinal biopsies and study approval

Biopsies were collected from human whole intestinal transplants from multi-organ donors. All donors were healthy without any indication for intestinal diseases. Donor 1 was female, Caucasian, 46 years, donor 2 was male, Hispanic, 23 years and donor 3 was male, Caucasian, 36 years. Biopsies of the descending colon were used to generate respective organoid lines. Due to small sample size the influence of sex on our model was not considered. However, samples covered both sexes.

Organs unsuitable for transplantation were procured from donors via Novobiosis, Inc. (Research Triangle Park, Durham, North Carolina, USA). For organ acquisition approval by the Organ Procurement Organizations (OPO) was followed, adhering to consent and deidentification guidelines set by both the OPOs and the United Network for Organ Sharing (UNOS) within the US Transplantation Network framework. Immediate family members of the donors provided permission for organ donations. During the whole donation process guidelines of the federal organizations UNOS and the Federal Drug Administration (FDA) were followed.

The study was executed adhering to the principles of the WMA Declaration of Helsinki and the Department of Health and Human Services Belmont Report.

#### Organoid line generation

Organoid line generation from descending colon biopsies and maintenance was performed as previously published.^22^^,^^23^^,^^61^ In short, crypts were isolated after incubation in 10 mM EDTA in PBS for 1 h at 4°C and then embedded in Matrigel (Corning). Organoids were passaged every 4–5 days at a ratio of 1:6-1:10 by triturating 10 times in 6 mL base medium (Table S1) after a 45 s incubation in Accutase (Gibco) followed by centrifugation at 150*g* for 5 min. Organoids were resuspended in Matrigel and seeded in 45 μL drops in a 24-well plate. After >30 min incubation in a humidified incubator at 37°C with 5% CO_2_ 500 μL expansion medium (Table S1) was added containing Y-27632 for the first 2–3 days of culture. Medium was changed every 2–3 days.

#### Cell lines

R-Spondin 1 conditioned medium was produced by HEK293T cells stably transfected with the mouse R-spondin1 protein tagged with C-terminus HA and N-terminus Fc. Cells were tested negative regularly for Mycoplasma. Cell line was not authenticated, but presence of R-Spondin 1 in conditioned medium was confirmed by ELISA. Conditioned medium was produced after selection of cells in DMEM with 10% FCS containing 300 μg/mL Zeocin by growing the confluent cells for 7 days in advanced DMEM/F12 supplemented with 1x GlutaMAX, 10 mM HEPES and 50 mg/mL Primocin.

### Method details

#### Chip establishment and differentiation

A detailed protocol for all following steps can be found elsewhere.^61^ Shortly, 1.8 μL of neutralized 5 mg/mL bovine Collagen I (Advanced Biomatrix) was added to the gel channel of one chip of the OrganoPlate 3-lane 40 (Mimetas) and polymerized at 37°C for 60 min. Then 100 μL growth medium (Table S1) was added to the bottom channel followed by the addition of 25 μL coating solution, 25 μg/mL human Collagen IV (Sigma), to the top outlet. After overnight incubation at 37°C medium and coating solution was replaced by 50 μL expansion medium containing Y-27632. Single cell suspension was generated from organoid culture by incubation with Accutase for 90 s followed by triturating 20 times before adding 20000 cells in 2 μL expansion medium to the top inlet. The plate was then placed at a 70° angle for 4 h at 37°C to allow cell settling against the gel before returning it to 0° angle. After overnight incubation the medium was replaced with growth medium and transferred to an interval shaker set to 7°/8 min. Medium was replaced every 2–3 days. For ML generation medium was changed to expansion medium on day 6. Cell expansion and tube formation was assessed by brightfield imaging (Nikon).

#### TEER measurements

Throughout the culturing period TEER values were accessed using the OrganoTEER device (Mimetas) according to the manufacturer’s protocol. In short, the chip was placed in the OrganoTEER device and recordings were performed at 37°C with 5% CO_2_. Recorded values were normalized to the growth area.

#### Permeability assay

On day 10 of culture medium was removed and 20 μL pre-warmed medium was added to the gel and bottom in- and outlet respectively. Then 70 μL medium containing 0.5 mg/mL FITC-dextran 4 kDa and TRITC-dextran 70 kDa was added to the top channel. Localization of the dye was followed by fluorescence microscopy with a time-lapse of 30 min/5 min intervals using a fluorescence microscope (Nikon).

#### Immunohistochemistry

Cells were fixed on day 10 of culture by replacing the medium with 4% PFA. After 20–30 min at 4°C cells were washed with PBS, permeabilized with 0.3% Triton X-100 for 10 min and blocked with 2% FBS/2% BSA/0.1% Tween 20 in PBS for >30 min at room temperature. Then primary antibody solution was added, anti-ZO-1 (1:100; Life Technologies, 61–7300), anti-Occludin (1:100; Life Technologies, 33–1500), anti-MUC2 (1:200; Santa Cruz Biotechnology, sc-515032), anti-KI67 (1:200; abcam, ab15580), and incubated for 2 h at room temperature. After washing secondary antibody, anti-rabbit-488 (1:500; Invitrogen, A11034) or anti-mouse-568 (1:500; Invitrogen, A11031), Phalloidin-643 (1:500; ATTO TEC, AD 643-81) and Hoechst 33342 (1:500; Fisher Scientific, H3570) were added and incubated for 1 h at room temperature. The chips were washed and then maintained with PBS at 4°C until imaging using confocal microscopy (Nikon).

#### Image analysis KI67 and MUC2

For the analysis of KI67 and MUC2 a z stack was acquired containing the basal and apical cell surface within a range of 10–20 μm and 1.68 μm between each image. For the quantification of KI67^+^ and MUC2^+^ cells a maximum intensity projection image was generated, and positive cells were identified. Total cell number was acquired using Fiji including BioVOXXEL plugin with StarDist.^51^^,^^52^^,^^60^ For intensity distribution analysis, the mean intensity for all acquired z stack images was calculated. Then the image with the highest nuclei staining intensity was used as reference to center all images independent of cell height. The mean intensity for the respective staining was then normalized to the number of nuclei to account for variable cell numbers and plotted with reference to the nuclei. The area under the curve was determined to calculate statistical significance.

#### Stimulation and sampling

Digested gliadin (PT-gliadin) was prepared as previously described.^62^^,^^63^ Shortly, 4 g gliadin from wheat (Sigma-Aldrich) and 80 mg pepsin (Sigma-Aldrich) were dissolved in 40 mL 0.2 N HCl incubated for 2 h at 37°C under constant agitation. The pH was adjusted to 7.4 with 2 N NaOH before the addition of 80 mg trypsin (Sigma-Aldrich) and a 4-h incubation at 37°C. The suspension was centrifuged at 750 g for 5 min and the supernatant sterile filtered. The control was prepared in a similar fashion without the addition of gliadin.

On day 10 of culture medium was removed from each chip and replaced with the respective medium. The chips were then exposed to 100 ng/mL Flagellin (Invitrogen), 100 ng/mL LPS (Sigma-Aldrich), 2 mg/mL PT-gliadin or 10 ng/mL TNF-α (PeproTech), 10 ng/mL IL-1β (PeproTech) and 10 ng/mL IFN-γ (PeproTech) for 4 h. The supernatant from the luminal and basal compartment was collected and stored at −20°C until further analysis.

#### Cytokine release assay

Cytokine concentrations in the collected supernatant were analyzed using an 11-panel bead-based multiplex assay (BioLegend) following the manufacturer’s suggestions. In total 5000 events of bead population A + B were recorded using a flow cytometer (Aria Fusion, BD). Based on standard curves individual cytokine concentrations were calculated using the respective data analysis software (BioLegend). Cytokine levels below the detection limit were considered as half the detection limit for representation and statistical analysis. In order to depict cytokine levels correlated to mRNA expression a min-max normalization was performed for each cytokine respectively.

#### RNA isolation and sequencing

For RNA isolation medium from the chips was removed on day 10 of culture and 50 μL RLT plus buffer containing β-Mercaptoethanol was added to the top in- and outlet respectively. After 5 min incubation the lysis buffer was transferred to a tube and total RNA was isolated using the RNeasy Plus Micro Kit (Qiagen) following the manufacturer’s protocol. Quantity was assessed using Qubit High Sensitivity Assay (Invitrogen). Bulk RNA Sequencing was performed by Novogene using an Illumina Novaseg HiSeq Pair-Ended 150 bp, with a sequencing depth of 9 G, equaling 30 million reads per sample.

#### Bulk RNA-seq data analysis

Bulk RNA-seq data underwent preprocessing using the nf-core/rnaseq pipeline (version 3.8.1).^57^ The GRCh38 reference genome with genome annotation version GRCh38 (release 106, Ensembl) was used for read alignment using STAR, and gene expression levels were quantified via Salmon.^58^^,^^59^ For the differential expression analysis DESeq2 was used and only genes were considered for inclusion in the analysis if they exhibit more than 1 count-per-million in the compared groups.^53^ A significance threshold of adjusted *p* value (FDR) < 0.05 was applied to identify genes showing significant differential expression. The donor was included in the generalized linear model as explanatory variable accounting for donor variations. Contrasts were extracted from differentiation as the main effect of interest (ML vs. CL). Gene set enrichment analysis was performed by employing a hypergeometric test implemented in the hypeR package, based on gene sets sourced from the Molecular Signatures Database (MSigDB).^54^^,^^55^^,^^56^ A threshold of adjusted *p* value (FDR) < 0.05 was employed to consider the terms as significantly enriched.

### Quantification and statistical analysis

#### Statistics

Data is represented as mean with standard deviation. Groups were compared using unpaired *t* test and *p* values below 0.05 considered as significant. If more than two groups were compared an ordinary one-way ANOVA with Šídák’s multiple comparison test was performed comparing the indicated groups. Significance is represented as ∗ = 0.05, ∗∗ = 0.01, ∗∗∗ = 0.001 and ∗∗∗∗ = 0.0001. Detailed information can be found in the respective figure legend. Outliers were identified using ROUT outlier test (Q = 1%). All statistical analyses not related to bulk RNA-seq analysis were performed using GraphPad Prism 9 Software (GraphPad Software, Inc.). Experiments were randomized and blinded whenever possible. Biorender was used for the graphical abstract.

## Supplemental information

Document S1. Figures S1–S4 and Table S1

Table S2. Differentially expressed genes identified in ML vs. CL condition, related to Figure 4

## References

[1] Fujii M., Matano M., Toshimitsu K., Takano A., Mikami Y., Nishikori S., Sugimoto S., Sato T. (2018). Human Intestinal Organoids Maintain Self-Renewal Capacity and Cellular Diversity in Niche-Inspired Culture Condition. Cell Stem Cell.

[2] Villenave R., Wales S.Q., Hamkins-Indik T., Papafragkou E., Weaver J.C., Ferrante T.C., Bahinski A., Elkins C.A., Kulka M., Ingber D.E. (2017). Human gut-on-a-chip supports polarized infection of coxsackie B1 virus in vitro. PLoS One.

[3] Jalili-Firoozinezhad S., Gazzaniga F.S., Calamari E.L., Camacho D.M., Fadel C.W., Bein A., Swenor B., Nestor B., Cronce M.J., Tovaglieri A. (2019). A complex human gut microbiome cultured in an anaerobic intestine-on-a-chip. Nat. Biomed. Eng..

[4] Trietsch S.J., Naumovska E., Kurek D., Setyawati M.C., Vormann M.K., Wilschut K.J., Lanz H.L., Nicolas A., Ng C.P., Joore J. (2017). Membrane-free culture and real-time barrier integrity assessment of perfused intestinal epithelium tubes. Nat. Commun..

[5] Gjorevski N., Avignon B., Gérard R., Cabon L., Roth A.B., Bscheider M., Moisan A. (2020). Neutrophilic infiltration in organ-on-a-chip model of tissue inflammation. Lab Chip.

[6] Gijzen L., Marescotti D., Raineri E., Nicolas A., Lanz H.L., Guerrera D., van Vught R., Joore J., Vulto P., Peitsch M.C. (2020). An Intestine-on-a-Chip Model of Plug-and-Play Modularity to Study Inflammatory Processes. SLAS Technol..

[7] Beaurivage C., Naumovska E., Chang Y.X., Elstak E.D., Nicolas A., Wouters H., van Moolenbroek G., Lanz H.L., Trietsch S.J., Joore J. (2019). Development of a gut-on-a-chip model for high throughput disease modeling and drug discovery. Int. J. Mol. Sci..

[8] Nicolas A., Schavemaker F., Kosim K., Kurek D., Haarmans M., Bulst M., Lee K., Wegner S., Hankemeier T., Joore J. (2021). High throughput transepithelial electrical resistance (TEER) measurements on perfused membrane-free epithelia. Lab Chip.

[9] Workman M.J., Gleeson J.P., Troisi E.J., Estrada H.Q., Kerns S.J., Hinojosa C.D., Hamilton G.A., Targan S.R., Svendsen C.N., Barrett R.J. (2018). Enhanced Utilization of Induced Pluripotent Stem Cell–Derived Human Intestinal Organoids Using Microengineered Chips. Cmgh.

[10] Naumovska E., Aalderink G., Valencia C.W., Kosim K., Nicolas A., Brown S., Vulto P., Erdmann K.S., Kurek D. (2020). Direct on-chip differentiation of intestinal tubules from induced pluripotent stem cells. Int. J. Mol. Sci..

[11] Moerkens R., Mooiweer J., Ramírez-Sánchez A.D., Oelen R., Franke L., Wijmenga C., Barrett R.J., Jonkers I.H., Withoff S. (2024). An iPSC-derived small intestine-on-chip with self-organizing epithelial, mesenchymal, and neural cells. Cell Rep..

[12] Kasendra M., Luc R., Yin J., Manatakis D.V., Kulkarni G., Lucchesi C., Sliz J., Apostolou A., Sunuwar L., Obrigewitch J. (2020). Duodenum intestine-chip for preclinical drug assessment in a human relevant model. eLife.

[13] Kasendra M., Tovaglieri A., Sontheimer-Phelps A., Jalili-Firoozinezhad S., Bein A., Chalkiadaki A., Scholl W., Zhang C., Rickner H., Richmond C.A. (2018). Development of a primary human Small Intestine-on-a-Chip using biopsy-derived organoids. Sci. Rep..

[14] Sontheimer-Phelps A., Chou D.B., Tovaglieri A., Ferrante T.C., Duckworth T., Fadel C., Frismantas V., Sutherland A.D., Jalili-Firoozinezhad S., Kasendra M. (2020). Human Colon-on-a-Chip Enables Continuous In Vitro Analysis of Colon Mucus Layer Accumulation and Physiology. Cmgh.

[15] Shin W., Ambrosini Y.M., Shin Y.C., Wu A., Min S., Koh D., Park S., Kim S., Koh H., Kim H.J. (2020). Robust Formation of an Epithelial Layer of Human Intestinal Organoids in a Polydimethylsiloxane-Based Gut-on-a-Chip Microdevice. Front. Med. Technol..

[16] Mitrofanova O., Nikolaev M., Xu Q., Broguiere N., Cubela I., Camp J.G., Bscheider M., Lutolf M.P. (2024). Bioengineered human colon organoids with in vivo-like cellular complexity and function. Cell Stem Cell.

[17] Lorenzo-Martín L.F., Broguiere N., Langer J., Tillard L., Nikolaev M., Coukos G., Homicsko K., Lutolf M.P. (2025). Patient-derived mini-colons enable long-term modeling of tumor–microenvironment complexity. Nat. Biotechnol..

[18] Beaurivage C., Kanapeckaite A., Loomans C., Erdmann K.S., Stallen J., Janssen R.A.J. (2020). Development of a human primary gut-on-a-chip to model inflammatory processes. Sci. Rep..

[19] Apostolou, A. A Micro-engineered Human Colon Intestine-Chip Platform to Study Leaky Barrier Athanasia. 10.1101/2020.08.28.271759.

[20] Apostolou A., Panchakshari R.A., Banerjee A., Manatakis D.V., Paraskevopoulou M.D., Luc R., Abu-Ali G., Dimitriou A., Lucchesi C., Kulkarni G. (2021). A novel microphysiological colon platform to decipher mechanisms driving human intestinal permeability. Cell. Mol. Gastroenterol. Hepatol..

[21] Nikolaev M., Mitrofanova O., Broguiere N., Geraldo S., Dutta D., Tabata Y., Elci B., Brandenberg N., Kolotuev I., Gjorevski N. (2020). Homeostatic mini-intestines through scaffold-guided organoid morphogenesis. Nature.

[22] Sato T., Stange D.E., Ferrante M., Vries R.G.J., Van Es J.H., Van Den Brink S., Van Houdt W.J., Pronk A., Van Gorp J., Siersema P.D., Clevers H. (2011). Long-term expansion of epithelial organoids from human colon, adenoma, adenocarcinoma, and Barrett’s epithelium. Gastroenterology.

[23] Hensel I.V., Éliás S., Steinhauer M., Stoll B., Benfatto S., Merkt W., Krienke S., Lorenz H.M., Haas J., Wildemann B., Resnik-Docampo M. (2024). SLE serum induces altered goblet cell differentiation and leakiness in human intestinal organoids. EMBO Mol. Med..

[24] Burclaff J., Bliton R.J., Breau K.A., Ok M.T., Gomez-Martinez I., Ranek J.S., Bhatt A.P., Purvis J.E., Woosley J.T., Magness S.T. (2022). A Proximal-to-Distal Survey of Healthy Adult Human Small Intestine and Colon Epithelium by Single-Cell Transcriptomics. Cmgh.

[25] Harnack C., Berger H., Liu L., Mollenkopf H.-J., Strowig T., Sigal M. (2023). Short-term mucosal disruption enables colibactin-producing E. coli to cause long-term perturbation of colonic homeostasis. Gut Microbes.

[26] Johansson M.E.V., Hansson G.C. (2016). Immunological aspects of intestinal mucus and mucins. Nat. Rev. Immunol..

[27] Hayashi F., Smith K.D., Ozinsky A., Hawn T.R., Yi E.C., Goodlett D.R., Eng J.K., Akira S., Underhill D.M., Aderem A. (2001). The innate immune response to bacterial flagellin is mediated by Toll-like receptor 5. Nature.

[28] Shimazu R., Akashi S., Ogata H., Nagai Y., Fukudome K., Miyake K., Kimoto M. (1999). MD-2, a molecule that confers lipopolysaccharide responsiveness on toll- like receptor 4. J. Exp. Med..

[29] Lammers K.M., Khandelwal S., Chaudhry F., Kryszak D., Puppa E.L., Casolaro V., Fasano A. (2011). Identification of a novel immunomodulatory gliadin peptide that causes interleukin-8 release in a chemokine receptor CXCR3-dependent manner only in patients with coeliac disease. Immunology.

[30] Tsukita S., Furuse M., Itoh M. (2001). Multifunctional strands in tight junctions. Nat. Rev. Mol. Cell Biol..

[31] Brunner J., Ragupathy S., Borchard G. (2021). Target specific tight junction modulators. Adv. Drug Deliv. Rev..

[32] Helander H.F., Fändriks L. (2014). Surface area of the digestive tract-revisited. Scand. J. Gastroenterol..

[33] Hu J.C.E., Weiß F., Bojarski C., Branchi F., Schulzke J.D., Fromm M., Krug S.M. (2021). Expression of tricellular tight junction proteins and the paracellular macromolecule barrier are recovered in remission of ulcerative colitis. BMC Gastroenterol..

[34] Wilms E., Troost F.J., Elizalde M., Winkens B., de Vos P., Mujagic Z., Jonkers D.M.A.E., Masclee A.A.M. (2020). Intestinal barrier function is maintained with aging – a comprehensive study in healthy subjects and irritable bowel syndrome patients. Sci. Rep..

[35] Sato T., Vries R.G., Snippert H.J., Van De Wetering M., Barker N., Stange D.E., Van Es J.H., Abo A., Kujala P., Peters P.J., Clevers H. (2009). Single Lgr5 stem cells build crypt-villus structures in vitro without a mesenchymal niche. Nature.

[36] Noel G., Baetz N.W., Staab J.F., Donowitz M., Kovbasnjuk O., Pasetti M.F., Zachos N.C. (2017). A primary human macrophage-enteroid co-culture model to investigate mucosal gut physiology and host-pathogen interactions. Sci. Rep..

[37] Wang Y., Kim R., Hwang S.H.J., Dutton J., Sims C.E., Allbritton N.L. (2018). Analysis of Interleukin 8 Secretion by a Stem-Cell-Derived Human-Intestinal-Epithelial-Monolayer Platform. Anal. Chem..

[38] Kayisoglu O., Weiss F., Niklas C., Pierotti I., Pompaiah M., Wallaschek N., Germer C.T., Wiegering A., Bartfeld S. (2021). Location-specific cell identity rather than exposure to GI microbiota defines many innate immune signalling cascades in the gut epithelium. Gut.

[39] Lee A.Y.S., Eri R., Lyons A.B., Grimm M.C., Korner H. (2013). CC Chemokine Ligand 20 and Its Cognate Receptor CCR6 in Mucosal T Cell Immunology and Inflammatory Bowel Disease: Odd Couple or Axis of Evil?. Front. Immunol..

[40] Yang C.C., Ogawa H., Dwinell M.B., McCole D.F., Eckmann L., Kagnoff M.F. (2005). Chemokine receptor CCR6 transduces signals that activate p130^Cas^ and alter cAMP-stimulated ion transport in human intestinal epithelial cells. Am. J. Physiol. Cell Physiol..

[41] Szabady R.L., McCormick B.A. (2013). Control of Neutrophil Inflammation at Mucosal Surfaces by Secreted Epithelial Products. Front. Immunol..

[42] Herfarth H.H., Martin C.F., Sandler R.S., Kappelman M.D., Long M.D. (2014). Prevalence of a gluten-free diet and improvement of clinical symptoms in patients with inflammatory bowel diseases. Inflamm. Bowel Dis..

[43] Goel G., Daveson A.J.M., Hooi C.E., Tye-Din J.A., Wang S., Szymczak E., Williams L.J., Dzuris J.L., Neff K.M., Truitt K.E., Anderson R.P. (2020). Serum cytokines elevated during gluten-mediated cytokine release in coeliac disease. Clin. Exp. Immunol..

[44] Lettesjö H., Hansson T., Bergqvist Å., Grönlund J., Dannaeus A. (2004). Enhanced interleukin-18 levels in the peripheral blood of children with coeliac disease. Clin. Exp. Immunol..

[45] Wright C.W., Li N., Shaffer L., Hill A., Boyer N., Alves S.E., Venkataraman S., Biswas K., Lieberman L.A., Mohammadi S. (2023). Establishment of a 96-well transwell system using primary human gut organoids to capture multiple quantitative pathway readouts. Sci. Rep..

[46] Martin K.R., Wong H.L., Witko-Sarsat V., Wicks I.P. (2021). G-CSF – A double edge sword in neutrophil mediated immunity. Semin. Immunol..

[47] Malashchenko V.V., Meniailo M.E., Shmarov V.A., Gazatova N.D., Melashchenko O.B., Goncharov A.G., Seledtsova G.V., Seledtsov V.I. (2018). Direct anti-inflammatory effects of granulocyte colony-stimulating factor (G-CSF) on activation and functional properties of human T cell subpopulations in vitro. Cell. Immunol..

[48] Meshkibaf S., Martins A.J., Henry G.T., Kim S.O. (2016). Protective role of G-CSF in dextran sulfate sodium-induced acute colitis through generating gut-homing macrophages. Cytokine.

[49] Meshkibaf S., Martins A.J., Henry G.T., Kim S.O. (2016). Protective role of G-CSF in dextran sulfate sodium-induced acute colitis through generating gut-homing macrophages. Cytokine.

[50] Hinman S.S., Wang Y., Kim R., Allbritton N.L. (2021). In vitro generation of self-renewing human intestinal epithelia over planar and shaped collagen hydrogels. Nat. Protoc..

[51] Schindelin J., Arganda-Carreras I., Frise E., Kaynig V., Longair M., Pietzsch T., Preibisch S., Rueden C., Saalfeld S., Schmid B. (2012). Fiji: an open-source platform for biological-image analysis. Nat. Methods.

[52] Brocher, J. (2022). biovoxxel/BioVoxxel-Toolbox: BioVoxxel Toolbox. 10.5281/ZENODO.5986130.

[53] Love M.I., Huber W., Anders S. (2014). Moderated estimation of fold change and dispersion for RNA-seq data with DESeq2. Genome Biol..

[54] Federico A., Monti S. (2020). HypeR: An R package for geneset enrichment workflows. Bioinformatics.

[55] Liberzon A., Subramanian A., Pinchback R., Thorvaldsdóttir H., Tamayo P., Mesirov J.P. (2011). Molecular signatures database (MSigDB) 3.0. Bioinformatics.

[56] Dolgalev I. msigdbr: MSigDB Gene Sets for Multiple Organisms in a Tidy Data Format. R package version 25.1.1. https://igordot.github.io/msigdbr/.

[57] Patel, H., Ewels, P., Peltzer, A., Hammarén, R., Botvinnik, O., Sturm, G., Moreno, D., Vemuri, P., silviamorins, Pantano, L., et al. (2022). nf-core/rnaseq: nf-core/rnaseq v3.8.1 - Plastered Magnesium Mongoose. https://doi.org/10.5281/ZENODO.6587789.

[58] Patro R., Duggal G., Love M.I., Irizarry R.A., Kingsford C. (2017). Salmon provides fast and bias-aware quantification of transcript expression. Nat. Methods.

[59] Dobin A., Davis C.A., Schlesinger F., Drenkow J., Zaleski C., Jha S., Batut P., Chaisson M., Gingeras T.R. (2013). STAR: Ultrafast universal RNA-seq aligner. Bioinformatics.

[60] Schmidt U., Weigert M., Broaddus C., Myers G., Frangi A.F., Schnabel J.A., Davatzikos C., Alberola-López C., Fichtinger G. (2018). Cell Detection with Star-Convex Polygons BT - Medical Image Computing and Computer Assisted Intervention – MICCAI 2018.

[61] Hensel I.V., Steinhauer M., Fairless R., Resnik-Docampo M. (2024). Protocol for generating and analyzing organ-on-chip using human and mouse intestinal organoids. STAR Protoc..

[62] Drago S., El Asmar R., Di Pierro M., Grazia Clemente M., Tripathi A., Sapone A., Thakar M., Iacono G., Carroccio A., D’Agate C. (2006). Gliadin, zonulin and gut permeability: Effects on celiac and non-celiac intestinal mucosa and intestinal cell lines. Scand. J. Gastroenterol..

[63] Gagliardi M., Clemente N., Monzani R., Fusaro L., Ferrari E., Saverio V., Grieco G., Pańczyszyn E., Carton F., Santoro C. (2021). Gut-Ex-Vivo system as a model to study gluten response in celiac disease. Cell Death Discov..

