# Supplementary material for: Advanced human organoid-on-chip with physiological cellular complexity reveals bidirectional secretion patterns
Source: iScience. 2025 Dec 15;29(2):114418. doi: 10.1016/j.isci.2025.114418 (PMC12829128; doi:10.1016/j.isci.2025.114418)
Supplement: Document S1. Figures S1–S4 and Table S1 [file mmc1.pdf]

iScience, Volume *29*

Supplemental information

Advanced human organoid-on-chip with physiological cellular complexity reveals bidirectional secretion patterns

Inga Viktoria Hensel, Szabolcs Éliás, Michelle Steinhauer, Claudia Günther, and Martín Resnik-Docampo

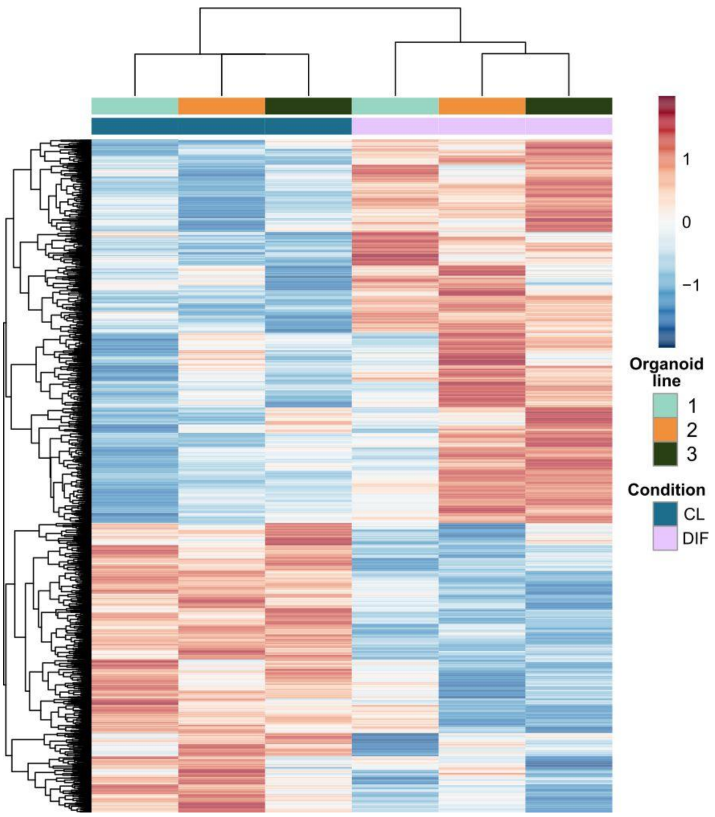

Figure S1: Multi-lineage phenotype in OoC, related to Figure 4.
Heatmap of all differentially expressed genes (p_{adj} <0.05).

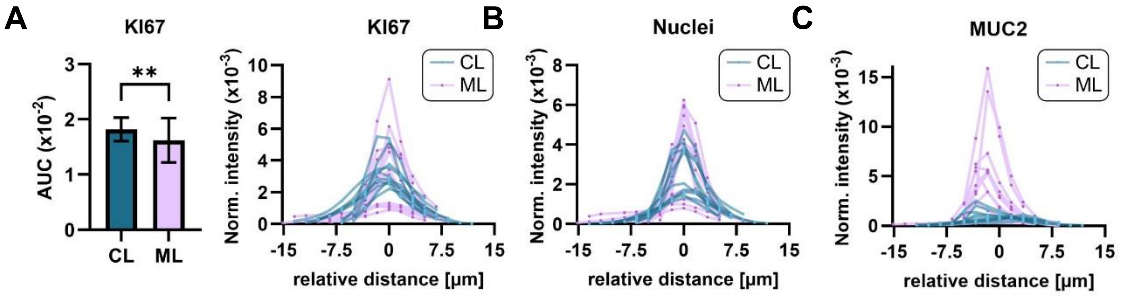

Figure S2: Distinct cell-type composition of in vitro intestinal epithelial barrier, related to Figure 4.
(A) Quantification of KI67 distribution along the z-axis. Area under the curve (AUC) was analyzed to compare the mean intensity distribution of KI67. The mean intensity was normalized to the total number of nuclei.
(B) Quantification of nuclei staining distribution along the z-axis. The mean intensity was normalized to the total number of nuclei. The maximum intensity was used to center all analyzed images.
(C) Quantification of MUC2 distribution along the z-axis. Area under the curve (AUC) was analyzed to compare mean intensity distribution of MUC2. The mean intensity was normalized to the total number of nuclei.
Each dot represents one chip n=10-15. Two-three donors in two independent experiments were analyzed. Data is shown as mean ±SD. Statistical significance was determined using t-test and significance is represented as * = 0.05, ** = 0.01, *** = 0.001 and **** = 0.0001.

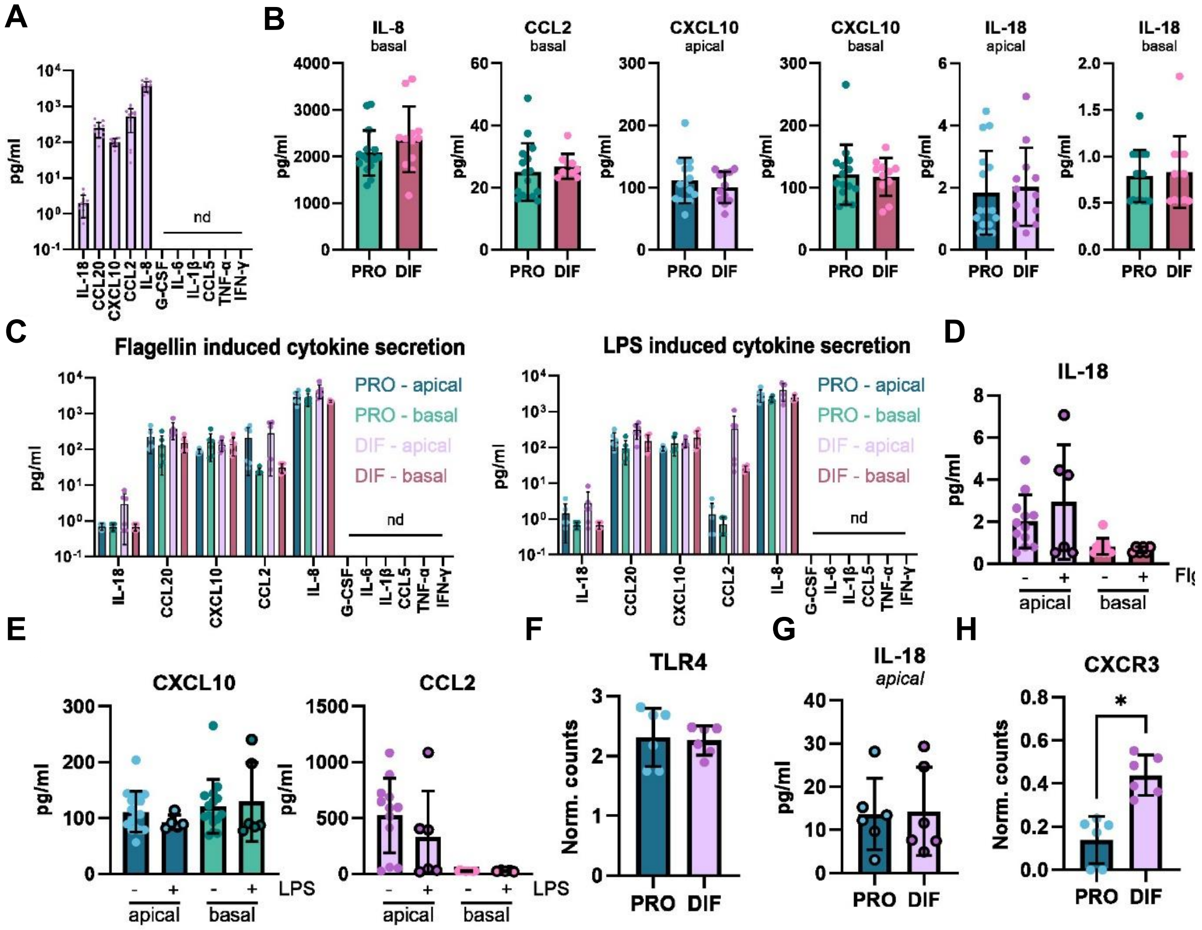

Figure S3: Cell type-dependent cytokine release under basal and stimulated conditions, related to Figure 5.

(A) Absolute values of measured cytokine concentration in the luminal compartment of ML condition are shown. Cytokine concentrations that were below the detection limit are shown as not detectable (nd).

(B) Cytokine concentrations in the luminal and basal compartment of unstimulated CL and ML condition are shown as indicated.

(C) Graphical overview of all cytokine concentrations for all measured cytokines are shown for luminal and basal compartment of CL and ML condition upon Flagellin stimulation. Cytokine concentrations that were below the detection limit are shown as not detectable (nd).

(D) IL-18 concentrations in luminal and basal compartment of ML condition after Flagellin (Flg) stimulation and the respective controls are shown.

(E) Graphical overview of all cytokine concentrations for all measured cytokines are shown for luminal and basal compartment of CL and ML condition upon LPS stimulation. Cytokine concentrations that were below the detection limit are shown as not detectable (nd).

(F) CXCL10 and CCL2 concentrations in luminal and basal compartment of CL and ML condition respectively after LPS stimulation and the respective controls are shown.

(G) Gene expression of TLR4, identified as differentially expressed gene in RNA-seq analysis, is shown as normalized counts. Data represents 3 donors in n=2 replicates.

(H) Gene expression of CXCR3, identified as differentially expressed gene in RNA-seq analysis, is shown as normalized counts. Data represents 3 donors in n=2 replicates.

All measurements were performed on day 10 of culture after 4 h stimulation. Cytokine data is shown for 2 donors, n=12-16 for controls and n=6 for stimulation and digestion controls. All data is shown as mean with SD. Statistical significance was determined using B: unpaired t-test, G, H: paired t-test and E, F: one-way ANOVA with Šídák's multiple comparison. Significance is represented as * = 0.05, ** = 0.01, *** = 0.001 and **** = 0.0001.

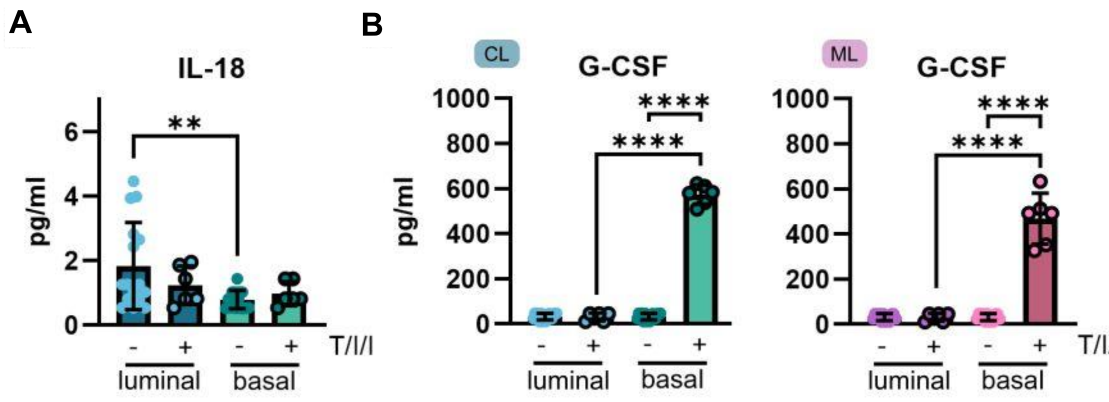

Figure S4: Cytokine release upon stimulation with proinflammatory cocktail, related to Figure 6.
(A) IL-18 concentrations in luminal and basal compartment of CL condition after stimulation with TNF-α (T), IL-1β (I) and IFN-γ (I) and the respective controls (-) are shown.
(B) G-CSF concentrations in luminal and basal compartment of CL and ML condition after stimulation with TNF-α (T), IL-1β (I) and IFN-γ (I) and the respective controls (-) are shown.
All measurements were performed on day 10 of culture after 4 h stimulation. Cytokine data is shown for 2 donors, n=12-16 for controls and n=6 for stimulation. All data is shown as mean with SD. Statistical significance was determined one-way ANOVA with Šídák's multiple comparison. Significance is represented as * = 0.05, ** = 0.01, *** = 0.001 and **** = 0.0001.

Table S1: Medium composition, related to STAR methods.

	Base medium	Expansion medium	Growth medium
advanced DMEM/F12			
R-Spondin1-conditioned medium (stably transfected HEK293T cells)		1x	1x
B27		1x	1x
HEPES	10 mM	10 mM	10 mM
GlutaMAX	1x	1x	1x
Nicotinamide			10 mM
Noggin		100 ng/ml	100 ng/ml
N-Acetyl-Cysteine		1 mM	1.25 mM
Primocin	100 µg/ml	100 µg/ml	100 µg/ml
surrogate Wnt		0.5 nM	0.5 nM
EGF		50 ng/ml	50 ng/ml
IGF-1		100 ng/ml	
FGF-2		50 ng/ml	
SB202190			10 µM
A83-01		500 nM	500 nM
Leu[15]-Gastrin I		10 nM	10 nM
CHIR99021			
Valproic acid			
Y-27632		10 µM	10 µM
